# Improved shuffled frog leaping algorithm on system reliability analysis

**DOI:** 10.1186/s40708-019-0095-4

**Published:** 2019-01-31

**Authors:** Yancang Li, Zhen Yan

**Affiliations:** 1 College of Water Conservancy and Hydroelectric Power, Hebei University of Engineering, Handan, China; 2 College of Civil Engineering, Hebei University of Engineering, Handan, China

**Keywords:** Hybrid optimization method, Shuffled frog leaping algorithm, Bacterial foraging algorithm, Levy flight, System reliability

## Abstract

With the increase in system complexity, the intelligent heuristic optimization methods have received more and more attention on system reliability analysis. However, the objective functions and constraint conditions of system reliability are nonlinear. Thereby, a hybrid optimization method was proposed, based on the shuffled frog leaping algorithm and bacterial foraging algorithm, to solve the problem of system reliability and redundancy allocation. First, random grouping strategy was added to maintain the diversity of the population. Then, the Levy flight update strategy was used to increase the global search ability. Finally, the method of migration operation was introduced to escape from local optimums. The proposed methodology, a new version of the SFLA algorithm, was then applied to the mathematical test and the operation of the system reliability model, respectively. Results show that compared to the common methods, it can obtain the best solution, with the maximum value of the system reliability.

## Introduction

The system reliability refers to the system ability of performing the required function under prescribed conditions and within the stipulated time. A complex system generally contains several connected components which are parallel connection and serial connection or neither. The reliability directly affects the performance of the system. If the index of reliability cannot meet the corresponding requirements, the possibility of malfunction will be higher when the system is more complex and it will cause the greater damage. And the complex system reliability optimization is aimed to obtain the highest reliability with seeking a best design scheme in some resource-constraint conditions, or to achieve maximum economic benefits and minimizing investment with meeting the requirements of a certain reliability index. While considerable advances have been made in system reliability, the study in system reliability, especially in the problem of redundancy allocation, has been dull. And there is still a huge space for development. In order to make sure the low failure rate, the key point is to find an efficient and precise method to calculate the optimal allocation problems. Therefore, the direction how to optimize the complex system reliability has become a foremost research topic.

In complex systems, it is not so easy to find an outstanding reliability optimization approach which can completely express the system reliability and redundancy allocation. The redundancy allocation generally allows normal operation when the instruments, equipment or others are in the abnormal situation. Many studies have been made to explore the approaches. In recent years, a lot of researches also tried to find another method to analyze the system reliability. Ditlevsen and Bjerager [1] proposed the thought of modeling structural systems which consisted of series and parallel systems. What’s more, with considering the uncertain factors, a versatile reliability-based design optimization approach was put forward for system design, which can compute the probabilities of unsatisfactory performance at both component and system levels [2]. Youn and Wang pointed out two primary challenges about system reliability evaluation and proposed a complementary intersection event, which could develop the complementary intersection method (CIM) for system reliability analysis with high efficiency and accuracy [3]. The objective functions and constraint conditions of complex system reliability are nonlinear [4]. It tends to be the non-differentiable, discontinuity, multidimensional and highly nonlinear NP-hard problem with constraint condition. And the objective functions usually have more than one local extremum. It is difficult to work out the results of these problems and hard to reach the global optimal by using the traditional methods. Thus, the appearance and development of intelligent algorithm have been provided a new tool to solve the problem of system reliability optimization.

To some extent, these algorithms can solve the complex system reliability optimization problems. But there are still some limitations, and we still have a long way to go. Therefore, it is vital to explore the effective solving methods.

Nowadays, hybrid optimization algorithms have become popular solution methods for the nonlinear model. Coit and Smith early used a combined neural network and GA approach to solve the NP-hard problem. The GA played the role of obtaining the minimum cost solution by selecting the appropriate components for a series–parallel system, and a neural network was applied to assess the system reliability value [5]. Lobato et al. analyzed the reliability-based optimization by using differential evolution and inverse reliability analysis for engineering system design. And the double-loop model was used to improve the algorithm. Simulation test on several examples with different complexity shows that heuristic algorithm was more accurate than classical algorithm [6]. Gholizadeh and other scholars analyzed the Bayesian estimation for the electronic system and pointed out that if the faults were independent, E-Bayesian algorithm can improve the accuracy of analysis [7]. Zhang et al. [8] analyzed the multi-scale security system for micro-mini smart ammunition. Coccon et al. [9] proposed a new approach for system reliability analysis of offshore structures by using dominant failure modes identified by selective searching technique.

The great development of the meta-heuristic optimization techniques represents the impetus for utilizing shuffled frog leaping algorithm to identify the unknown parameters of the single-diode PV model [10]. It consisted of a combination of phenomenon-mimicking algorithms and mathematical techniques, the hybrid shuffled frog leaping algorithm [11]. The shuffled frog leaping algorithm was used to determine the number of clusters and the optimal kernel parameter. Compared with other swarm intelligence optimization methods, its main advantage is high-speed convergence where it combines the merits of both GA-based technique and the social behavior of the particle swarm optimization (PSO) approach [12]. Moreover, Orouji et al. indicated that the shuffled frog leaping algorithm has the best capability and most efficiency among other well-developed algorithms such as the genetic algorithm (GA), harmony search (HS), particle swarm optimization and simulated annealing (SA), with 3.97, 0.03, 0.33 and 0.08% improvement in obtained objective function values, respectively [13, 14].

In this paper, a newly hybrid optimization method was considered for the estimation of system reliability. Results were then compared with those obtained by other existing techniques in the case study.

## System reliability model

**Fig. 1:**
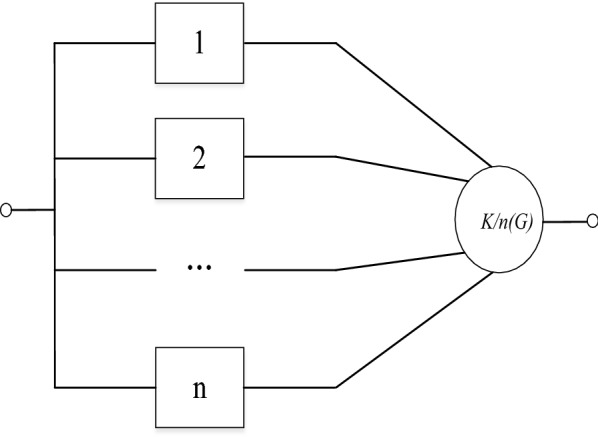
Reliability block diagram of *k*/*n*(*G*) system

Series systems, parallel systems and mixed systems are the three mainly representative systems. Herein, we focus on the series systems. A series system consists of many components that the failure of one component can cause the failure of the entire system or the whole system can be in normal working only if the whole component can work. And the reliability block diagram is shown in Fig. 1.

We denote the components by $C_{1} ,C_{2} , \ldots ,C_{n}$. Correspondingly, their reliabilities were denoted by $R_{1} ,R_{2} , \ldots ,R_{n}$. If the states of the components were considered as independence and the numerical value does not change over time, the system reliability is:

1 $$R_{s} = \prod\limits_{i = 1}^{n} {R_{i} } .$$

Here, we can clearly recognize the reliability of series system is always less than or equal to the reliability of the most unreliable components. And we can know $R_{s} \le \hbox{min} \left\{ {R_{i} } \right\}$. Thus, the high reliability of components should be chosen to reduce the number of series components in the phase of designing series system.

In the early stage of system design, one of the important parts is to determine the system reliability index of the composition unit based on the system reliability index of the whole system [15]. In the system design phase, it is easy to solve the problem about the system design if only considering the reliability index. However, the situation does not exist in actual engineering. Recognizing the relationship of each optimization index in the system could be the basis of the system design optimization. And the system reliability, cost, weight and volume are most commonly used. In this study, therefore, we only considered the four parts to measure the system reliability. The detail descriptions will be narrated in the fourth part.

## Improved hybrid optimization method

### Basic shuffled frog leaping algorithm

First proposed by Eusuff and Lansey [16], the hybrid shuffled frog leaping algorithm is an artificial algorithm based on the shuffled complex evolution and PSO methods. The shuffled frog leaping algorithm is a population-based heuristic optimization algorithm with cooperative search metaphor inspired by natural memetics. The algorithm uses memetic evolution in the form of influencing of ideas from one individual to another in a local search by simulating the food searching of frogs. The swamp has a number of stones at discrete locations where frogs can leap to find a stone with the maximum amount of food. The frogs are allowed to communicate with one another, so that they can improve their memes by using others’ information. Improvement in a meme is achieved when a frog that is far from the stone with a maximum amount of food leaps toward a frog closer to the food [17]. This leap results in altering the faraway frog’s position.

The hybrid shuffled frog leaping algorithm starts the optimization process by generating a set of frogs as the set of solutions in the first iteration. These frogs have a dimension that is equal to the number of decision variables. For example, in a problem with $N$ decision variables, the frogs are vectors ($X_{l} = \left[ {x_{l,1} ,x_{l,2} , \ldots ,x_{l,N} } \right]$). An objective function is then calculated for each frog and is used as the comparison criterion to compare the worth of each frog.

Next, frogs are sorted according to the calculated objective function using the descending or ascending method in a minimization or maximization problem, respectively [13]. Afterward, the frogs are divided into $K$ memeplexes. Thus, if the number of frogs is $M$, $M/K$ frogs will be located in each memeplex. In this process, the first sorted frog with the best obtained objective function moves toward the first memeplex, and the second sorted frog moves toward the second memeplex. This process continues until the *M*th sorted frog moves to the *M*th memeplex. In the next step, the (*M *+ 1)th frog moves toward the first memeplex, etc. By considering two best and worst frogs in each memeplex ($X_{{K{\text{b}}}}$ and $X_{{K{\text{w}}}}$) and the best global frog in the set of frogs $X_{\text{g}}$, the hybrid shuffled frog leaping algorithm tries to improve $X_{{K{\text{w}}}}$ in each iteration, by using Eqs. (2)–(4):

2 $$D_{K}^{t} = {\text{rand}}\left( {X_{{K{\text{b}}}} - X_{{K{\text{w}}}} } \right)$$

3 $$D_{\text{Min}} \le D_{{_{K} }}^{t} \le D_{\text{Max}}$$

4 $$X_{{K{\text{w}}}}^{t + 1} = X_{{K{\text{w}}}}^{t} + D_{K}^{t} ,$$

where $D_{K}^{t}$ is the change of the worst frog in *K*th memeplex in *t*th iteration; rand is a random value between 0 and 1; $D_{\text{Min}}$ and $D_{\text{Max}}$ are the minimum and maximum allowable values for the change of the worst frog, respectively; and $X_{{K{\text{w}}}}^{t}$ and $X_{{K{\text{w}}}}^{t + 1}$ are the position of the worst frog in *K*th memeplex in the *t*th and (*t *+ 1)th iterations, respectively. In this step, $X_{{K{\text{w}}}}^{t + 1}$ should be improved compared to the $X_{{K{\text{w}}}}^{t}$ even by generating a random new worst frog instead of $X_{{K{\text{w}}}}^{t}$.

### Improvement in the basic hybrid optimization

Application of the improved algorithm on system reliability is summarized in the following steps:

Step 1: Initialize. Select $m$ and $n$, where $m$ is the number of memeplexes and *n* is the number of frogs in each memeplex. Therefore, the total sample size $F$, in the swamp, is given by $F = mn$.

Step 2: Generate a virtual population. Sample $F$ virtual frogs $U\left( 1 \right),U\left( 2 \right), \ldots ,U\left( F \right)$ in the feasible space. The *i*th frog is represented as a vector of decision variable values $U\left( i \right) = \left( {U_{i}^{1} ,U_{i}^{2} , \ldots ,U_{i}^{kd} } \right),$ that is, a candidate solution containing $K$ cluster centers.

Step 3: Rank frogs. Sort the $F$ frogs in order of decreasing performance value. Store them in an array $X = \left\{ {U\left( i \right),f\left( i \right),i = 1, \ldots ,F} \right\}$ so that $i = 1$ represents the frog with the best performance value. Record the best frog’s position $P_{X}$, in the entire population ($F$ frogs; where $P_{X} = U\left( 1 \right)$).

Step 4: Partition frogs into memeplex. Partition array $X$ into $Y_{1} ,Y_{2} , \ldots ,Y_{m}$, each containing $n$ frogs.

Step 5: Memetic evolutions within each memeplex. Evolve each memeplex $Y^{l} ,l = 1, \ldots ,m$.

After partitioning frogs to $m$ memeplexes, evolve each memeplex and each of them should iterate $N$ times. After the memeplexes have been evolved, the algorithm returns to the global exploration for shuffling. Below are the details of the local search for each memeplex.

Local exploration: NSFLA algorithm.

Step 5.1 Set $im = 0$, where $im$ counts the number of memeplexes and will be compared with the total number $m$ of memeplexes. Set $iN = 0$, where $iN$ counts the number of evolutionary steps and will be compared with the maximum number $N$ of steps to be completed within each memeplex. Within each memeplex, the frogs with the best and the worst fitness are identified as $P_{\text{b}}$ and $P_{\text{w}}$, respectively. Also, the frog with the global best fitness is identified as $P_{\text{g}}$. Then, an evolution process is applied to improve only the frog with the worst fitness (i.e., not all frogs) in each cycle.

Step 5.2 Set $$im = im + 1$$.

Step 5.3 Set $$iN = iN + 1$$.

Step 5.4 Improve the worst frog’s position. The position of the frog with the worst fitness is adjusted as follows:

Change in frog position

5 $$D_{i} = {\text{rand}}(m) \times \left( {P_{\text{b}} - P_{\text{w}} } \right)$$

New position

6 $$\begin{aligned} P_{\text{w}} = & {\text{current}}\;{\text{position}};P_{\text{w}} + D_{i} \\ & \left( {D_{{\rm max} } \ge D_{i} \ge - D_{{\rm max} } } \right), \\ \end{aligned}$$

where ${\text{rand}}()$ is a random number between 0 and 1 and $D_{{\rm max} }$ is the maximum allowed change in a frog’s position.

Step 5.5 If this process produces a better frog (solution), it replaces the worst frog. Then, frogs are sorted according to the calculated objective function using the descending method. And the half better went on as predicted, and the half worse will update with Levy flight to increase the global search ability.

The location will be updated by Levy flight:

7 $$x_{i}^{{\left( {t + 1} \right)}} = x_{i}^{\left( t \right)} + \alpha \oplus {\text{Levy}}\left( \lambda \right) \quad i = 1,2, \ldots ,n$$

in which the $x_{i}^{\left( t \right)}$ is the $t$th location of $x_{i}$; $\oplus$ is the point-to-point multiplication; $\alpha$ is the parameter of step length control; ${\text{Levy}}\left( \lambda \right)$ is the random search path and has the limit:

8 $${\text{Levy}}\,\sim\,u = t^{ - \lambda } \quad 1 < \lambda \le 3.$$

The essence of Levy flight is a kind of method of random step length. And the step length conforms to Levy distribution. However, implementation is not realized currently due to the complex. Thus, it is commonly simulated through Mantegna algorithm. It can be described as follows:

A calculation formula for the step length is:

9 $$s = \frac{\mu }{{\left| v \right|^{1/\beta } }},$$

where the $\mu$ and $v$ are the Gaussian distribution which is defined as:

$$\begin{aligned} & \mu \,\sim\,N\left( {0,\sigma_{\mu }^{2} } \right) \\ & v\,\sim\,N\left( {0,\sigma_{v}^{2} } \right) \\ & \sigma_{\mu } = \left\{ {\frac{{\varGamma \left( {1 + \beta } \right)\sin \left( {\frac{\pi \beta }{2}} \right)}}{{\varGamma \left[ {\frac{{\left( {1 + \beta } \right)}}{2}} \right]\beta^{{2\frac{{\left( {\beta - 1} \right)}}{2}}} }}} \right\}^{1/\beta } \\ & \sigma_{v} = 1 \\ \end{aligned}$$

in which $\beta$ is commonly defined as 1.5 and $\varGamma$ is the standard Gamma function.

Therefore, the update location equation of Levy flight can be summarized as follows:

10 $$X_{g + 1,i} = X_{g,i} + \alpha \frac{\mu }{{\left| v \right|^{1/\beta } }}\left( {X_{g,i} - P_{\text{g}} } \right).$$

Step 5.6 The improved method of migration operation in BFA was introduced. In terms of the same fitness of the individuals, one individual will be operated by the adaptive migration in order to keep the diversity. And the fitness of the group will be sorted by using the descending method. And the distribution probability can be applied as follows:

11 $${\text{Ped}}\left( i \right) = 1 - \left( {\frac{S - i}{S - 1}} \right)^{2} ,$$

where $i$ is the individual serial number and $S$ is the population size.

It can be seen from Eq. (11) that the probability migration of the best individual is 0, and namely, the best individual will not migrate. And the lower the fitness of the individual is, the higher the migration probability is.

Step 5.7 If this process produces a better frog (solution), it replaces the worst frog. Otherwise, repeat.

Step 5.8 If no improvement becomes possible in this latter case, then a new solution is randomly generated to replace the worst frog with another frog having any arbitrary fitness.

Step 5.9 If $iN < N$, go to step 5.3.

Step 5.10 If $im < m$, go to step 5.2. Otherwise, return to the global search to shuffle memeplexes.

Step 6: Shuffle memeplexes. After a defined number of memetic evolutionary steps within each memeplex, replace $Y_{1} , \ldots ,Y_{m}$ into $X$ such that $X = \left\{ {Yk,k = 1, \ldots ,m} \right\}$. Sort $X$ in order of decreasing performance value. Update the population the best frog’s position $P_{X}$.

Step 7: Check convergences. If the convergence criteria are satisfied, stop. Otherwise, return to step 4. Typically, the decision on when to stop is made by a prespecified number of consecutive time loops when at least one frog carries the “best memetic pattern” without change. Alternatively, a maximum total number of function evaluations can be defined.

## Simulation and test

In order to verify the convergence, speed and accuracy of the hybrid algorithm, the five simulation tests are carried out. In this paper, we selected standard hybrid shuffled frog leaping algorithm (SFLA) and compared four kinds of other algorithms, such as BFA, SFLA [17] and PSO-SFLA [14]. And the test environment is: Windows 7, 4G memory, 2.5 GHz. Table 1 shows the details of these test functions, where $f\left( {x_{1} ,x_{2} , \ldots ,x_{n} } \right)$ = objective function for $x_{1}$ to $x_{i}$ decision variables; *i *= counter for dimension; and *n *= total number of dimensions.

**Table 1:** Definition of the selected unconstrained and constrained test function

Test function	Formula	Search domain	Global optimum
$f_{1}$ Sphere	$f\left( {x_{1} , \ldots ,x_{n} } \right) = \sum\limits_{i = 1}^{n} {x_{i}^{2} }$	$- \,5 \le x_{i} \le + 5$	$f\left( {x_{1} , \ldots ,x_{n} } \right) = f\left( {0, \ldots ,0} \right) = 0$
$f_{2}$ Shubert	$f\left( {x_{1} , \ldots ,x_{n} } \right) = - \sum\limits_{i = 1}^{n} {\sum\limits_{j = 1}^{5} {j\sin \left[ {\left( {j + 1} \right)x_{i} + j} \right]} }$	$- 10 \le x_{i} \le + 10$	$f\left( {x_{1} , \ldots ,x_{n} } \right) = - 24.0625$
$f_{3}$ Rastrigin	$f\left( {x_{1} , \ldots ,x_{n} } \right) = \mathop \sum \limits_{i = 1}^{n} \left( {x_{i}^{2} - 10\cos \left( {2\pi x_{i} } \right) + 10} \right)$	$- 5.12 \le x_{i} \le + 5.12$	$f\left( {x_{1} , \ldots ,x_{n} } \right) = 0$
$f_{4}$ Rosenbrock	$f\left( {x_{1} , \ldots ,x_{n} } \right) = \mathop \sum \limits_{i = 1}^{n} \left( {100\left( {x_{x} - x_{i}^{2} } \right)^{2} + \left( {1 - x_{i} } \right)^{2} } \right)$	$- 2 \le x_{i} \le + 2$	$f\left( {x_{1} , \ldots ,x_{n} } \right) = f\left( {1, \ldots ,1} \right) = 0$
$f_{5}$ Easom	$f\left( {x_{1} ,x_{2} } \right) = - \cos \left( {x_{1} } \right) \cdot \cos \left( {x_{2} } \right) \cdot {\text{e}}^{{ - \left( {\left( {x_{1} - \pi } \right)^{2} + \left( {x_{2} - \pi } \right)^{2} } \right)}}$	$- 100 \le x_{i} \le + 100\;i = 1,2$	$f\left( {x_{1} ,x_{2} } \right) = f\left( {\pi ,\pi } \right) = - 1$

The $f_{1}$ Sphere is simple unimodal function to verify the rate of convergence. $f_{2}$ Shubert possesses 760 local minimum points. However, there are only 18 global minimum points, and the value is − 186.73. Within the range of (10, 10), its minimum value is − 24.062499. The function is able to fully test the ability of seeking the global optimal with jumping out the local extremum. $f_{3}$ Rastrigin is a typical nonlinear multimodal function which has a wide range of search space, tall obstacles and a lot of local minimum points. It is commonly considered to be difficult to deal with the complex multimodal problems. $f_{4}$ Rosenbrock is a classic complex optimization function which the function of each contour is roughly parabolic, and the global minimum value is also located in the valley of parabolic. It is easy to find the valley, but it is tough to find the global minimum because the change of value is small in the valley. The critical point is that the global optimal value is located in a smooth and narrow parabolic in the valley. $f_{5}$ Easom is a nonlinear function which is hard to obtain the optimal solution. Because the global minimum solution distributes in the very narrow, its peripheral is almost flat.

## Engineering application

In this section, a case study will be applied to validate and compare the performance of the proposed hybrid optimization method with three other algorithms. In the process of prefabricated building design and construction, there are mainly four parts, such as design, manufacture, transportation and construction. The target is realizing the high system reliability and the low cost. Herein, a prefabricated house will be built, and there are four cost limits which consist of design cost $C_{\text{d}}$, manufacture cost $C_{\text{m}}$, transportation cost $C_{\text{t}}$ and construction cost $C_{\text{c}}$. And the main system goal of optimal design can be described as: seeking the optional design subsystem $\alpha_{1} ,\alpha_{2} ,\alpha_{3} ,\alpha_{4}$ and the subsystem redundancy $m_{1} ,m_{2} ,m_{3} ,m_{4}$ to ensure the highest system reliability $R$ with the limitation of the whole cost. The model can be expressed as follows:

12 $$\begin{aligned} R\left( \delta \right)_{{\rm max} } & = R\left( {m,\alpha } \right) = \prod\limits_{i = 1}^{n} {R_{i} } \left( {m_{i} ,\alpha_{i} } \right) \\ & = R_{1} \left( {m_{1} ,\alpha_{1} } \right) + R_{2} \left( {m_{2} ,\alpha_{2} } \right) \\ & \quad + R_{3} \left( {m_{3} ,\alpha_{3} } \right) + R_{4} \left( {m_{4} ,\alpha_{4} } \right) \\ \end{aligned}$$

13 $$\begin{aligned} C_{\text{d}} \left( {m,\alpha } \right) = & \sum\limits_{i = 1}^{4} {c_{{{\text{d}}i}} } \left( {\alpha_{{{\text{d}}i}} } \right)m_{{{\text{d}}i}} \le C_{{{\text{d}}{\rm max} }} \\ C_{\text{m}} \left( {m,\alpha } \right) = & \sum\limits_{i = 1}^{4} {c_{{{\text{m}}i}} } \left( {\alpha_{{{\text{m}}i}} } \right)m_{{{\text{m}}i}} \le C_{{{\text{m}}{\rm max} }} \\ C_{\text{t}} \left( {m,\alpha } \right) = & \sum\limits_{i = 1}^{4} {c_{{{\text{t}}i}} } \left( {\alpha_{{{\text{t}}i}} } \right)m_{{{\text{t}}i}} \le C_{{{\text{t}}{\rm max} }} \\ C_{\text{c}} \left( {m,\alpha } \right) = & \sum\limits_{i = 1}^{4} {c_{{{\text{c}}i}} } \left( {\alpha_{{{\text{c}}i}} } \right)m_{{{\text{c}}i}} \le C_{{{\text{c}}{\rm max} }} \\ \end{aligned}$$

in which $m_{i} \left( {i = 1,2,3,4} \right)$, and the other system parameter values are shown in Table 2:

**Table 2:** System parameter values

Subsystem $i$	Optional design											
	1				2				3			
	$C_{d}$	$C_{m}$	$C_{t}$	$C_{c}$	$C_{d}$	$C_{m}$	$C_{t}$	$C_{c}$	$C_{d}$	$C_{m}$	$C_{t}$	$C_{c}$
1	0.98	0.92	0.97	0.98	0.99	0.92	0.96	0.96	0.96	0.95	0.97	0.97
2	0.97	0.94	0.97	0.94	0.96	0.92	0.97	0.94	0.98	0.97	0.96	0.95
3	0.94	0.96	0.96	0.97	0.92	0.96	0.99	0.95	0.98	0.95	0.97	0.95
4	0.96	0.94	0.98	0.97	0.94	0.95	0.99	0.97	0.99	0.95	0.94	0.98

We can obtain the stable optimal solution after a lot of running as follows.

$$\delta = \left[ {\left( {2,1} \right),\left( {2,3} \right),\left( {3,2} \right),\left( {2,2} \right)} \right].$$

And we can obtain the detailed solutions as given in Table 3.

**Table 3:** Optional design of $\delta$ and redundancy allocation

Subsystem $i$	Optional design	Redundancy allocation	Subsystem reliability
1	1	2	1 − (1 − *X*)^2^ =
2	3	2	1 − (1 − *X*)^2^ =
3	2	3	1 − (1 − *X*)^2^ =
4	2	2	1 − (1 − *X*)^2^ =

Then, we can obtain the reliability of the whole system based on Eq. (3): $R\left( \delta \right) = 0.9989$. We can obtain:

14 $$\begin{aligned} C_{\text{d}} \left( {m,\alpha } \right) = & \sum\limits_{i = 1}^{4} {c_{{{\text{d}}i}} } \left( {\alpha_{{{\text{d}}i}} } \right)m_{{{\text{d}}i}} = xx \le C_{{{\text{d}}{\rm max} }} \\ C_{\text{m}} \left( {m,\alpha } \right) = & \sum\limits_{i = 1}^{4} {c_{{{\text{m}}i}} } \left( {\alpha_{{{\text{m}}i}} } \right)m_{{{\text{m}}i}} = xx \le C_{{{\text{m}}{\rm max} }} \\ C_{\text{t}} \left( {m,\alpha } \right) = & \sum\limits_{i = 1}^{4} {c_{{{\text{t}}i}} } \left( {\alpha_{{{\text{t}}i}} } \right)m_{{{\text{t}}i}} = xx \le C_{{{\text{t}}{\rm max} }} \\ C_{\text{c}} \left( {m,\alpha } \right) = & \sum\limits_{i = 1}^{4} {c_{{{\text{c}}i}} } \left( {\alpha_{{{\text{c}}i}} } \right)m_{{{\text{c}}i}} = xx \le C_{{{\text{c}}{\rm max} }} . \\ \end{aligned}$$

**Fig. 2:**
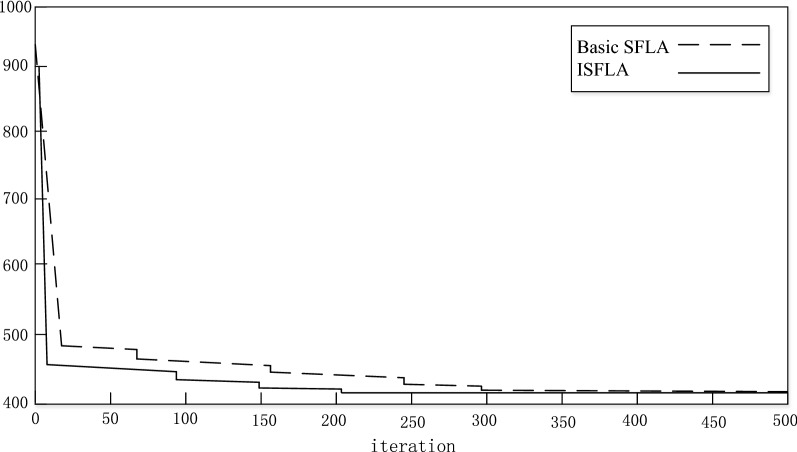
Comparison chart of TSP Eil51

Then, the iteration curves of system reliability which are calculated by basic SFLA and ISFLA are shown in Fig. 2.

From Fig. 2, we can see that, when the iteration reaches 200 runs, the system reliability will gradually increase. And the optimal solution will be stable. Although the running curve is not exactly the same due to the random of the algorithm, the most results will eventually reach the optimal solution.

## Conclusion

1. In the design of the building, it is necessary to consider the reliability of the system, especially for the prefabricated building with large-scale, complicated and systematic construction system. The mistakes of the construction will cause huge losses. To find a more effective algorithm to optimize the system reliability, an improved leapfrog algorithm was proposed. The results show that the proposed method is more accurate for structural reliability calculation. It provides a new idea and method for assembly research and reliability analysis.
2. Aiming at the shortcomings of the frog jump algorithm, an improved algorithm was proposed to enhance its calculation efficiency and accuracy.
3. The improved algorithm was applied to the standard frog jump algorithm to enhance the global and local search ability and improve the convergence speed.
4. As a new algorithm for the optimization problem, the hybrid shuffled frog leaping algorithm still has many imperfections. To improve the algorithm more effectively, for example, the parameter setting, we still have a long to go.
